# The Use of Neutron Analysis Techniques for Detecting The Concentration And Distribution of Chloride Ions in Archaeological Iron

**DOI:** 10.1111/arcm.12058

**Published:** 2013-10-17

**Authors:** D Watkinson, M Rimmer, Z Kasztovszky, Z Kis, B Maróti, L Szentmiklósi

**Affiliations:** Department of Archaeology and Conservation, Cardiff UniversityJohn Percival Building, Colum Drive, Cardiff, CF10 3EU, UK; Centre for Energy Research, Hungarian Academy of SciencesH-1525 Budapest, P.O. Box 49, Hungary

**Keywords:** Archaeological iron, PGAA, PGAI, Neutron tomography, Neutron radiography, Chloride, Corrosion, Conservation, Elemental analysis

## Abstract

Chloride (Cl) ions diffuse into iron objects during burial and drive corrosion after excavation. Located under corrosion layers, Cl is inaccessible to many analytical techniques. Neutron analysis offers non-destructive avenues for determining Cl content and distribution in objects. A pilot study used prompt gamma activation analysis (PGAA) and prompt gamma activation imaging (PGAI) to analyse the bulk concentration and longitudinal distribution of Cl in archaeological iron objects. This correlated with the object corrosion rate measured by oxygen consumption, and compared well with Cl measurement using a specific ion meter. High-Cl areas were linked with visible damage to the corrosion layers and attack of the iron core. Neutron techniques have significant advantages in the analysis of archaeological metals, including penetration depth and low detection limits.

## Introduction

Archaeological iron is damaged by chloride ions, which diffuse into the object during burial (Turgoose 1982, 1985). After excavation, oxidation forms ferrous chloride and akaganéite (β-FeOOH), which create significant pressure within the corrosion layers and cause cracking, fragmentation and break-up of objects (Turgoose 1982; Selwyn *et al*. 1999; Loeper-Attia 2007). Although the basic principles of this process are well understood, information regarding the three-dimensional distribution of chloride ions within the object and its relationship to patterns of cracking and the development of corrosion behaviour is not currently available. This is due to the difficulty in analysing for chloride ions, which are located deep within the object under the corrosion layers, and are not accessible to surface analytical techniques such as X-ray fluorescence, X-ray diffraction and Raman spectroscopy without cross-sectioning of objects or removal of overlying corrosion (Neff *et al*. 2004). These methods expose the interior of the object to oxygen, which could lead to oxidation of ferrous chloride (Réguer *et al*. 2007) and transformation of corrosion products prior to the analysis. Contamination from cutting and polishing is also a risk. Even if measures are taken to avoid these problems, through the use of non-aqueous lubricants and dry storage of the polished samples (Réguer *et al*. 2007; Rimmer and Wang 2010), surface analytical techniques are limited to a two-dimensional analysis, which does not account for the complex three-dimensional structure of the corrosion layers and metal core. Although analysis of bulk chloride is possible, this involves destruction of the object via digestion in order to liberate the chloride ions into solution for potentiometric/specific ion techniques (Watkinson 2010; Rimmer *et al*. 2013). It is therefore not suitable for valuable museum objects.

Non-invasive neutron techniques offer promising routes for analysis of archaeological and museum objects, but have only rarely been applied to archaeological iron (Selwyn and Argyropoulos 2006). Prompt gamma activation analysis (PGAA) is a non-destructive bulk analytical method, based on the detection of characteristic gamma-photons emitted in (n,γ) reactions (Révay *et al*. 2004) when a material is irradiated with a beam of cold neutrons from a nuclear research reactor. Since neutrons penetrate up to several centimetres deep into the material, PGAA provides bulk composition characteristic for the whole irradiated volume, which can be through the entire depth of a small object. Contrary to conventional neutron activation analysis (NAA), irradiation and detection are simultaneous. The energies and intensities of the peaks in the gamma spectra are independent of the chemical state of the material; hence the analytical result is free of matrix effects. In principle, PGAA is able to detect all the chemical elements except He, although sensitivity varies significantly, being dependent on the neutron capture cross-sections of the different nuclei. In most cases, major components and a few significant trace elements can be quantified. PGAA has previously been used on other archaeological and heritage objects; for example, to analyse bulk composition of silver coins (Kasztovszky *et al*. 2005), other metals (Kasztovszky *et al*. 2000) and stone artefacts (Kasztovszky *et al*. 2008).

In order to acquire spatially resolved elemental information, the Centre for Energy Research at the Budapest Neutron Centre has developed a unique combination of PGAA and neutron radiography (NR)/tomography (NT) (Belgya *et al*. 2008). NR or NT provides a 2D or 3D visual representation of the sample (Anderson *et al*. 2009), and the spatial data of internal regions can be linked to the position of a motorized sample stage (Kis *et al*. 2008). This allows the localization of areas of interest, which can then be moved accurately into the neutron beam, where the subsequent acquisition of gamma spectra results in characterization of the irradiated volume in a selective way. This makes the technique, called tomography/radiography-driven prompt gamma activation imaging (PGAI) (Belgya *et al*. 2008), feasible and productive. Some *a priori* knowledge about the object is often available, which can assist in selecting regions of interest. The use of collimators reduces the impinging neutron beam size from its maximum 20 × 20 mm^2^ to a minimum spot size of 5 mm^2^ to focus analysis in the desired areas. Neutron tomography at the same experimental station can be used to aid interpretation by giving a 3D visualization of the interior of the object which, for metal objects, can be superior to 2D X-radiography (Koleini *et al*. 2012).

A pilot study was developed under the European CHARISMA programme (http://www.charismaproject.eu/) to test the application of PGAA and PGAI for the analysis of Cl ion content and distribution within archaeological iron objects. The study had two aims: (a) to determine the comparability of PGAA analysis of bulk Cl with traditional methods of measuring chloride ions using digestion and specific ion measurement, and (b) to investigate the relationship between Cl distribution, corrosion morphology and patterns of damage caused by corrosion. This was part of a three-year Arts and Humanities Research Council (AHRC)/Engineering and Physical Sciences Research Council (EPSRC) Science and Heritage funded project at Cardiff University studying the prediction of corrosion behaviour of iron objects to determine how their heritage value is affected, and the effectiveness of measures to reduce the corrosion rate. The establishment of a clear link between the location and concentration of chloride within objects and the appearance of corrosion damage on the surface is an important step in understanding iron corrosion behaviour and predicting long-term survivability of objects in museum collections.

## Methodology

Objects first had their corrosion rate measured by oxygen consumption at Cardiff University. They were analysed at the Budapest Neutron Centre, then later digested at Cardiff University to measure their chloride ion content by the specific ion method.

### Samples

Objects for the analysis were selected from three different sites: Billingsgate in London (BWB83) is a Roman/medieval waterlogged site, excavated in 1983. Caerleon in South Wales (CPF08) is a Roman fortress site, excavated in 2008. Usk in South Wales (USK) is a Roman site, excavated in the 1960s. The nails were selected from a group that had previously undergone oxygen consumption tests at Cardiff University to determine their corrosion rate (see below). Individual nails were assessed for suitability for analysis based on shape and size to fit the sample holder at the PGAA and NIPS–NORMA stations. Objects that displayed signs of corrosion behaviour—such as surface cracks in the corrosion products, appearance of fresh corrosion products and fragmentation—were preferred, avoiding objects that were too fragile to withstand mounting in the sample holders. Objects were X-ray radiographed prior to testing, and photographed before and after the corrosion rate test using a 12 MP Pentax k-r SLR camera with a 100 mm macro lens to visually record corrosion damage to the objects during the corrosion period. In total, 13 nails were subjected to PGAA and/or PGAI (Table 1).

**Table 1:** Details of the objects and the analyses carried out in this study

Sample	Mass (g)	Length (to nearest mm)	Oxygen consumption rate (mbar day^−1^ g^−1^)	Analyses carried out		
				PGAA	PGAI	NT
BWB83_153	4.21	52	0.032 ± 0.003		*	
BWB83_163	4.95	32	0.007 ± 0.001	*		
BWB83_168	5.91	43	0.008 ± 0.001	*		
BWB83_169	6.32	32	0.019 ± 0.002	*		
CPF08_016	2.98	37	0.255 ± 0.014	*		
CPF08_031	7.02	35	0.152 ± 0.055	*		
CPF08_051	9.44	48	0.314 ± 0.060	*		
CPF08_054	6.89	53	0.184 ± 0.005		*	
CPF08_059	4.81	41	0.132 ± 0.021	*		
CPF08_062	6.77	38	0.204 ± 0.014	*	*	*
CPF08_063	7.27	46	0.183 ± 0.005	*	*	
CPF08_064	8.14	57	0.146 ± 0.004		*	
USK_001	4.07	39	0.001 ± 0.010 (N.S.)	*		

^*^Indicates type of analysis performed on the object listed. N.S. = oxygen consumption was not statistically different from zero.

### Oxygen consumption

The corrosion rate of the objects was measured using oxygen consumption to monitor the rate of corrosion reactions in controlled relative humidity (RH) and temperature. This method is currently being pioneered at Cardiff University with archaeological iron, although it has previously been used to measure corrosion rates of flat pieces of wrought iron (Matthiesen and Wonsyld 2010). Each object was enclosed in a sealed environment created using 250 cm^3^ food preservation jars with brass/rubber sealing discs and rings. Each jar included a fixed quantity of silica gel, conditioned in a climate chamber for at least 4 weeks at the desired RH, and a temperature/RH logger. Ruthenium-based sensor spots were adhered to the inside of the glass jars, and the jars placed in a Binder KBF240 climate chamber to control the temperature. Once sealed, the oxygen partial pressure in the jars was measured using a WPI OxyMini fibre-optic oxygen meter, which illuminates the sensor spot and measures fluorescence, which is proportional to oxygen concentration. Control jars were also measured and any decrease in oxygen over time subtracted from the jars containing samples. The leakage rate from the jars was measured using seven nitrogen-filled control jars, and was less than 0.012 mbar day^−1^; this figure is accounted for in the oxygen data (Table 1).

The objects in this study had their oxygen consumption measured at 20°C ± 0.5 and 70% RH ± 2 for up to 159 days. The linear rate was extracted from the data points by means of regression (rate = slope of the regression line), and normalized to the mass of the object in grams. This provides the oxygen consumption rate in mbar day^−1^ g^−1^ (Table 1). Since the objects have been excavated for 30 years (Billingsgate) and 4 years (Caerleon) respectively, it was thought that any significant transformations of metastable corrosion products that might occur in the atmosphere will have taken place. The absence of organic matter in the reaction vessels means that biodeterioration that would deplete oxygen via respiratory action of microbes will not occur. Reactive phases in rust layers such as maghemite (γ-FeOOH) can, immediately after wetting at normal corrosion potentials, balance metal dissolution by reduction of iron (III) oxides rather than by the normal O_2_ reduction (Stratmann and Hoffmann 1989). Wetting of the desiccated iron objects at 70% RH when they are initially introduced to the reaction vessel could involve this type of reaction, meaning that some metal oxidation would be unrecorded by the oxygen consumption technique. However, since all objects have been stored in desiccated silica gel, exposed to the same RH during testing and are of similar size, they have a degree of standardization, which means that impact of any reactive phases on oxygen consumption is expected to be similar in all cases. Additionally, the corrosion rate is reported as oxygen consumption per day and is not converted to loss of metal; it therefore offers a comparative measure of overall corrosion rate for these objects rather than an absolute statement of their metal loss. With these provisos, oxygen consumption is used to represent the corrosion rate.

### PGAA

PGAA analysis was carried out at the Budapest Neutron Centre in May 2012. The facility is described in Szentmiklósi *et al*. (2010). The thermal equivalent intensity of the beam is ≈ 10^8^ cm^−2^ s^−1^. For irradiation of the objects, an attenuated and collimated beam of cold neutrons with a 20 × 20 mm cross-section was applied to avoid the overload of the counting electronics. The prompt gamma spectra were collected using a precisely calibrated Compton-suppressed HPGe detector, and evaluated using the Hypermet-PC program (Révay *et al*. 2005). The quantitative analysis is based on the *k*_0_-principle, using the spectroscopic data libraries developed at the Budapest PGAA laboratory (Révay *et al*. 2004). The average composition of each irradiated volume was determined using the methods described by Révay (2009). Objects were placed in bags made of thin Teflon film for PGAA. No damage or significant induced radioactivity was produced in the objects during the analysis. Element identification is based on the precise determination of characteristic prompt-gamma lines, while the amount of a given element is proportional to the peak intensities. The detected gamma-ray intensity *A(E)* is directly proportional to the mass of a given element *m*, the analytical sensitivity *S* and the measurement time *t*; that is, *A(E)* = *mSt*.

The mass ratios of arbitrary elements *X* and *Y* are calculated from peak area ratios and sensitivity ratios,

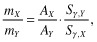
and will be independent of the actual amount of the sample and also of the exact neutron flux.

The sensitivities for the most intensive prompt-gamma lines of all chemical elements were determined by internal standardization measurements at the Budapest Research Reactor and have been collected in a new gamma-ray spectrum catalogue for PGAA by Révay *et al*. (2004).

### NR/NT-driven PGAI

A set-up called NORMA has been installed as a part of the NIPS experimental station at Budapest Neutron Centre (Szentmiklósi *et al*. 2013). The thermal equivalent flux of the guided cold neutron beam is about 2.7 × 10^7^ cm^−2^ s^−1^ and the cross-section of the neutron beam is 40 × 40 mm^2^. The neutrons transmitted through the properly positioned samples were detected by a two-dimensional position-sensitive detector (a ^6^Li-doped ZnS scintillator coupled to a Peltier-cooled CCD camera), located downstream of the sample. The spatial resolution of the imaging system ranges from 230 μm to 450 μm depending on the distance (0–100 mm, respectively) between the sample and the ZnS converter screen.

For the element analysis, the neutron beam was shaped to the desired size (either a 10 × 10 mm^2^ or 3 × 20 mm^2^ area) with collimators, to reach the optimal count rate of gamma-photons. The gamma-photons from the radiative neutron capture in the sample were detected with a Compton-suppressed HPGe detector. The solid angle of the gamma-ray detector was defined by a lead collimator, which had a size of 25 × 25 mm^2^. The typical acquisition time was about 1 h for each selected position. At present, elemental ratios can be determined with this technique. In this study, five objects were mounted vertically in a sample holder using Teflon strings, and the Cl/Fe ratios in the nails were analysed along the long axis, in steps of 10 mm or 3 mm depending on the size of collimator selected, to provide a longitudinal profile of Cl/Fe ratio. As the neutrons penetrate through the depth of the object, demonstrated by the radiography images, each analysis provides integral data for a horizontal slice of the object.

### Neutron tomography

At the same experimental station, one object was subjected to neutron tomography. Two-dimensional projected images were taken at predefined steps during a 180° rotation of a vertically mounted object. A total of 601 images were collected in successive angle increments of 0.3°. The filtered back-projection algorithm was applied to produce reconstructed slices of the object. Having stacked the slices using voxel rendering software, it is possible to create artificial cuts in any desired direction, and to visualize the object′s internal structure as slices or an animation.

### Specific ion meter

All of the objects that had been subjected to PGAA/PGAI at Budapest were subsequently digested whole in 5 M nitric acid (HNO_3_), according to previously published protocols (Rimmer 2010; Rimmer *et al*. 2012). Once digested, the samples were neutralized with 3 M NaOH, filtered to remove the ferrous precipitate and had their pH adjusted into the range 5–7. The chloride content was then measured using a calibrated Radiometer Analytical specific ion meter with a chloride-specific electrode and a mercury/mercury sulphate reference electrode. The detection limit is ∼1 mg l^−1^; precision is contextual and normally about 10%. The Cl concentration is reported as parts per million (ppm) in relation to the mass of the object to facilitate comparison between different objects (Watkinson 2010).

## Results

### PGAA

Bulk compositions of 10 corroded nails were determined (Table 2). Quantitative determination of elemental concentrations in wt% or in ppm units by PGAA requires the detection of all major elements. As oxygen is detected with very low sensitivity, its quantity can only be given by calculation using typical oxidation numbers of the elements. In this case, this would require that all metallic elements were fully oxidized (Révay 2009).

**Table 2:** Elemental composition of objects measured by PGAA: amounts of elements are given relative to the amount of Fe (m/m_Fe_); detection limits are given as m/m_Fe_; mean relative uncertainty is calculated from the relative uncertainties of each element across the 10 samples

	Element												
	H	B	Na	Al	Si	P	Cl	K	Ca	Ti	Mn	Sm	Gd
Detection limit (*m/m*_Fe_)	1.00E–03	4.29E–07	7.14E–04	1.43E–03	1.43E–03	7.14E–03	1.43E–05	1.29E–03	1.43E–03	7.14E–05	1.43E–04	1.43E–07	1.43E–07
Mean relative uncertainty (%)	1.4	1.6	2.8	3.8	5	11.4	2.3	5	7.5	7	3.8	5.5	11.1
BWB83_163	0.0019	1.89E–06	<DL	0.01	0.003	0.01	1.15E–04	<DL	0.004	1.00E–04	0.00084	<DL	<DL
BWB83_168	0.00109	1.25E–06	<DL	0.0023	0.002	<DL	7.15E–05	<DL	0.002	<DL	0.00048	<DL	<DL
BWB83_169	0.00118	1.45E–06	<DL	0.0049	0.005	<DL	9.42E–05	<DL	0.002	9.00E–05	0.00019	<DL	<DL
CPF08_016	0.0154	1.98E–05	0.0016	0.023	0.11	0.02	2.55E–03	0.004	0.005	1.10E–03	0.00058	1.80E–06	1.60E–06
CPF08_031	0.0204	3.34E–05	0.005	0.039	0.25	0.02	2.41E–03	0.0083	0.008	2.50E–03	0.00072	3.50E–06	4.00E–06
CPF08_051	0.0105	1.47E–05	<DL	0.016	0.041	0.01	2.96E–03	0.0016	0.006	4.30E–04	0.00037	4.30E–07	4.60E–07
CPF08_059	0.0104	1.33E–05	0.0011	0.018	0.069	0.02	6.43E–03	0.0029	0.004	8.00E–04	0.00048	1.10E–06	9.10E–07
CPF08_062	0.0101	1.10E–05	<DL	0.013	0.043	0.03	2.49E–03	0.0037	0.002	4.80E–04	0.00119	6.80E–07	5.20E–07
CPF08_063	0.0157	2.77E–05	<DL	0.016	0.058	0.02	5.30E–03	0.0022	0.007	6.90E–04	0.00064	9.40E–07	1.20E–06
USK_001	0.0264	4.55E–05	0.0027	0.033	0.17	0.03	8.74E–04	0.0094	0.025	1.80E–03	0.0014	2.50E–06	3.30E–06

The archaeological objects in this study, except USK_001, however, have a remaining metal core and corrosion layers in various states of oxidation, rendering this assumption untenable. Since oxygen cannot be measured directly and the degree of oxidation was not exactly known for the irradiated volume, the oxygen content was not determined by measurements or calculation. Therefore, only the elemental ratios can be considered in this study. Detection limits can be calculated according to Currie′s criterion (Currie 1968; Révay 2009), and can be expressed as mass ratios relative to iron, based on precise evaluation of a pair of peaks with similar energies. Mass ratios relative to Fe were quantitatively determined for H, B, Na, Al, Si, P, Cl, K, Ca, Ti, Mn, Sm and Gd, and are given in Table 2, together with detection limits and relative uncertainties.

Cl/Fe mass ratios determined by PGAA are characteristic for the Cl content of the irradiated volume, independent of oxygen content. In comparison with the specific ion measurements, the Cl/Fe ratio follows the same general pattern; however, there is some discrepancy in the results (Table 3). The Cl/Fe ratio derived from PGAA measurements for BWB83 objects underestimates the quantities of Cl as determined by the specific ion meter (see Discussion).

**Table 3:** Chloride data from PGAA and specific ion meter measurements

	Mass of object (g)	PGAA		Specific ion meter	
		Cl/Fe ratio	Cl/Fe ratio relative uncertainty (%)	Cl (mg)	Cl (ppm)*
BWB83_163	4.95	0.00011	3.30	2.44	493
BWB83_168	5.91	0.00007	3.95	2.92	494
BWB83_169	6.32	0.00009	3.89	1.44	228
CPF08_016	2.98	0.00255	1.87	5.37	1770
CPF08_031	7.02	0.00241	2.19	8.84	1266
CPF08_051	9.44	0.00296	2.22	9.46	1002
CPF08_059	4.81	0.00643	2.20	9.59	1988
CPF08_062	6.77	0.00249	1.88	7.42	1097
CPF08_063	7.27	0.00530	2.12	17.81	2459
USK_001	4.07	0.00087	1.96	1.54	379

^*^Cl (ppm) measured by specific ion meter is given in relation to the mass of the object (equivalent to mg kg^−1^ or μg g^−1^).

### PGAI

**Figure 1:**
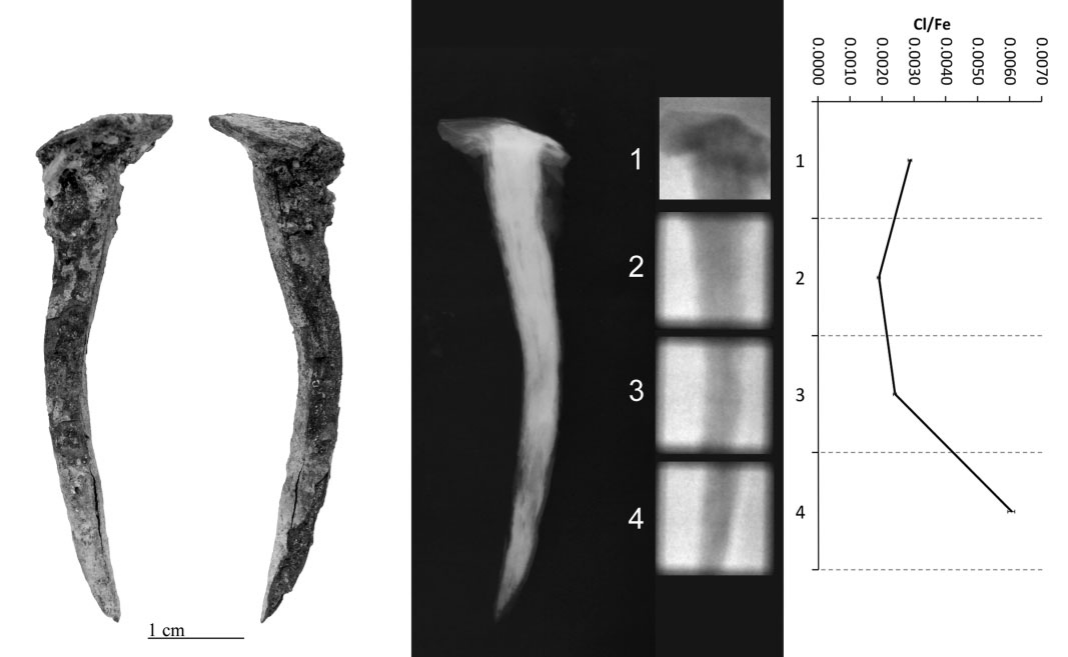
Left to right: a photograph, X-ray radiograph, neutron radiograph and the PGAI data for BWB83_153, using a 10 × 10 mm collimator.

**Figure 2:**
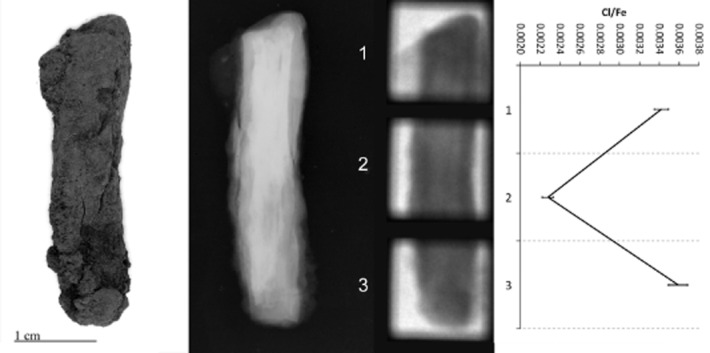
Left to right: a photograph, X-ray radiograph, neutron radiograph and the PGAI data for CPF08_062, using a 10 × 10 mm collimator.

**Figure 3:**
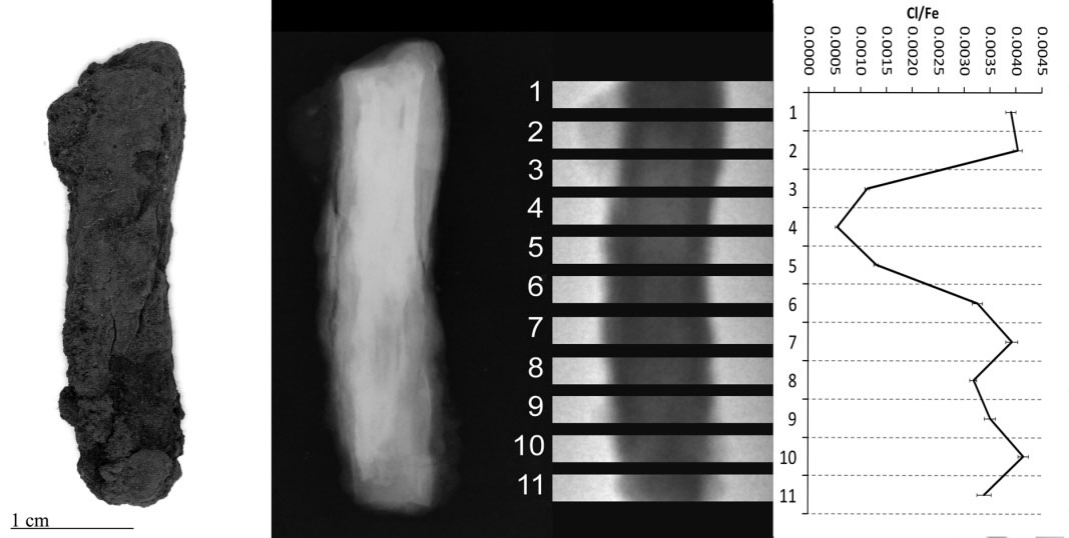
Left to right: a photograph, X-ray radiograph, neutron radiograph and the PGAI data for CPF08_062, using a 3 × 20 mm collimator.

**Figure 4:**
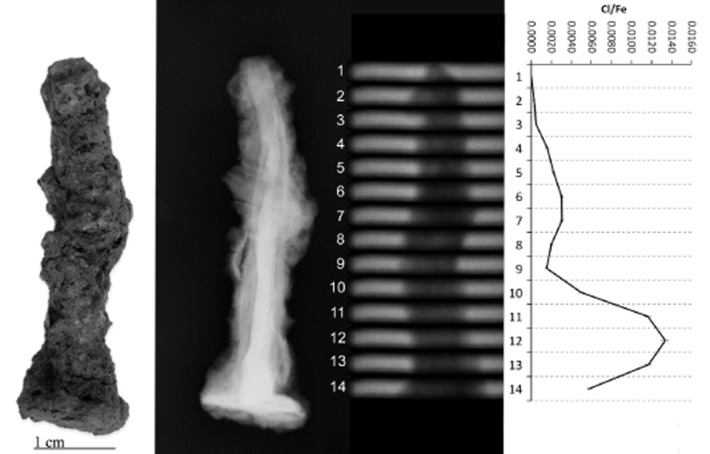
Left to right: a photograph, X-ray radiograph, neutron radiograph and the PGAI data for CPF08_063, using a 3 × 20 mm collimator.

**Figure 5:**
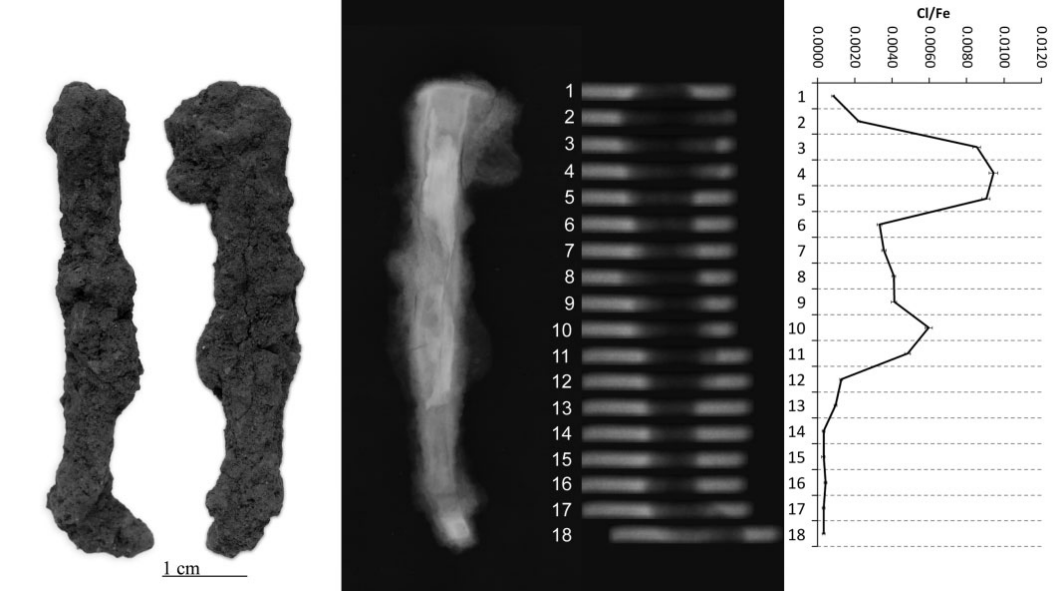
Left to right: a photograph, X-ray radiograph, neutron radiograph and the PGAI data for CPF08_064, using a 3 × 20 mm collimator.

PGAI data are shown in Figures 5, together with images and X-ray radiographs of the objects analysed. All display significant variability in Cl/Fe ratio along their length, from effectively zero to a maximum of 0.013 (CPF08_063, Fig. 4). Although the data from 10 mm blocks (Figs 1 and 2) is informative and shows some change in the distribution of Cl along the length of the object, the 3 mm data show the advantages of this analysis interval by the more informative and distinct Cl patterns visible (compare CPF08_062, where both 10 mm (Fig. 2) and 3 mm data (Fig. 3) were acquired).

CPF08_063 and 064 (Figs 4 and 5) have maximum Cl levels where high density (white) within the X-ray plate indicates remaining metal in the object, whereas low-density areas (grey–black) within the X-ray plates reveal fully mineralized sections of objects that tend to have negligible Cl content. CPF08_062 has high Cl at both ends (Fig. 3), where the radiograph shows attack of the metal along slag planes (vertical striations within the core) and extensive corrosion, with some metallic iron still evident. Similarly, BWB83_153 (Fig. 1) shows high Cl in the tip of the nail that has much corrosion, with a very large pit evident and residual metallic iron present.

For three out of five objects analysed, there is clear correlation between the areas of high Cl and visual damage phenomena on the surface. BWB83_153 (Fig. 1) has a large crack and spalling of the outer corrosion layer at the nail tip, corresponding to the highest Cl/Fe ratio. CPF08_062 (Fig. 3) and CPF08_054 have areas of corrosion product growth and flaking where there are peaks of Cl. For CPF08_063 (Fig. 4) and CPF08_064 (Fig. 5), there is less clear evidence of the physical manifestation of corrosion generally on their surface, except that CPF08_064 has a large crack in the central area, where there is a high Cl/Fe ratio, but does not show visual damage in the upper part, where the Cl level is even higher. There is no visual evidence of damage where the objects are totally mineralized, and this supports the theory that corrosion and its subsequent physical impact does not occur where no metal core remains. CPF08_063 appears relatively unaffected by corrosion in the area of high Cl around the head of the nail (Fig. 4), although there is a small patch of orange corrosion product in this area (not visible in the photograph), indicative of some active corrosion. This object experienced no significant alteration during the exposure to 70% RH during the corrosion test, despite its oxygen consumption being comparable to that of the other CPF08 objects (Table 1).

### Neutron tomography

**Figure 6:**
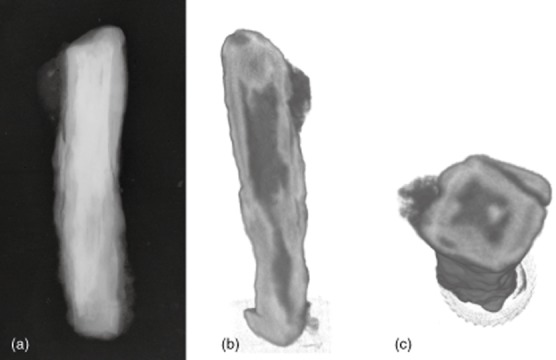
An X-radiograph of CPF08_062 (a), and a longitudinal cross-section (b) and a transverse cross-section (c) through the neutron tomography image of CPF08_062. Darker grey values represent higher neutron attenuation; that is, larger atomic density of Fe. This is due to the different densities of metallic iron and corrosion products.

Neutron tomographic imaging was carried out on only one object, CPF08_062, as an example to determine whether it provided useful additional information on the internal structure of the metal core and corrosion layers. Figure 6 shows two slices through the object and its X-radiograph. A comparison of the NT images with the two-dimensional X-ray image of this object shows that the 3D image and the ability to slice through the object in any desired direction does provide additional useful information. The metal core in the lower half is more contorted than is evident from the X-ray image (Figs 6 (a) and 6 (b)). The attack of the metal core in pits and along slag planes in the upper part of the object (Fig. 6 (c)), suggested by the X-ray, is more apparent in the 3D image. Different textures in the corrosion layers are also visible, showing the outline of the original shape of the object within the corrosion layer (Fig. 6 (c)).

## Discussion

### Comparison of two chloride measurement techniques

**Figure 7:**
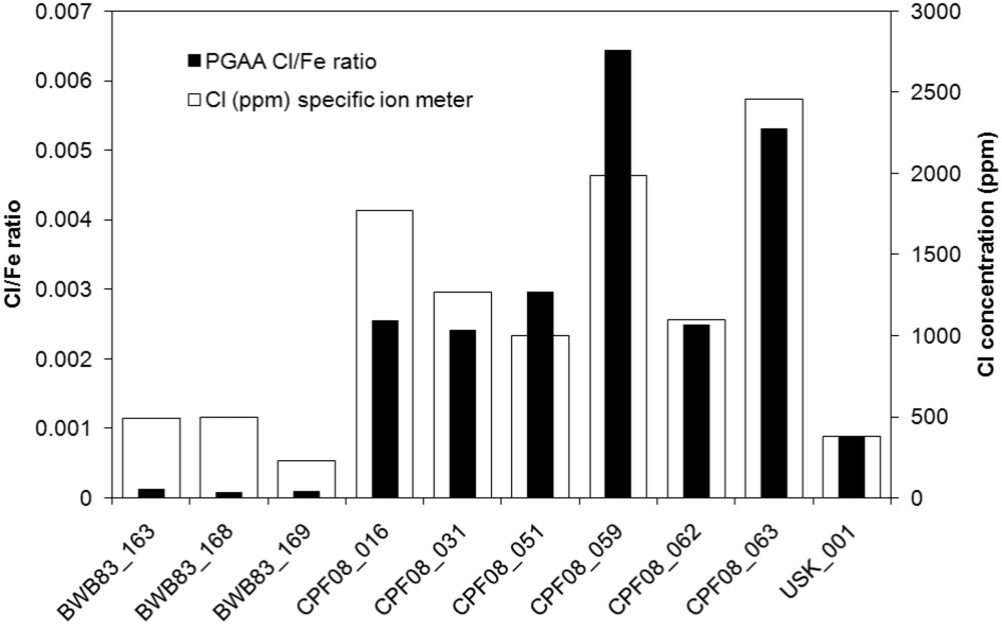
PGAA measurement and specific ion measurement of chloride (data from Table 3).

The first aim of the study was to determine whether PGAA measurement of Cl produces results comparable to those of the traditional method using a specific ion meter. As explained above, the presence of varying and unknown amounts of iron metal introduces some difficulties into the determination of absolute concentrations by PGAA, leading to the use of Cl/Fe ratios in this discussion. PGAA identified the same overall pattern in the data as the specific ion meter: BWB83 objects have much lower chloride contents than CPF08 objects (Fig. 7 and Table 3). Comparison with previous specific ion measurements of large numbers of digested objects from these sites shows that the chloride concentrations in these samples are within the expected range for each site, with the inland Roman Legionary fortress site at Caerleon producing higher concentrations of Cl in objects than the wetter and urban Billingsgate site (Rimmer 2010; Watkinson 2010; Rimmer *et al*. 2012).

**Figure 8:**
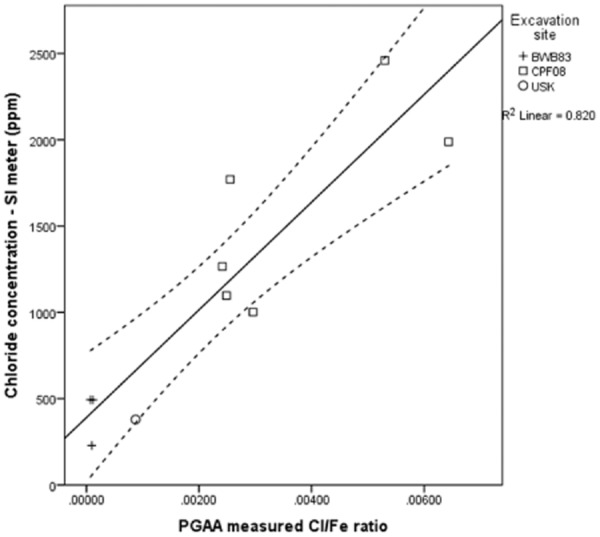
Correlation of the Cl/Fe ratio measured by PGAA and the Cl concentration measured by specific ion meter, with a linear regression line and a 95% confidence interval.

Figure 8 shows the correlation between Cl/Fe ratios and Cl measured by specific ion meter of the 10 objects measured by PGAA. There is a significant correlation between the two variables. Pearson′s correlation coefficient is 0.91 [*P* (one-tailed) < 0.01]. However, as neither variable is normally distributed and contains only 10 samples, non-parametric tests were also carried out to confirm the validity of the correlation. Significant correlations were obtained, though with lower coefficients (Spearman′s rho, *r*_s_ = 0.82, *P* (one-tailed) < 0.01; Kendall′s tau, *τ* = 0.60, *P* (one-tailed) < 0.05). Estimates of total chloride content within archaeological iron can be achieved in a non-invasive manner using linear regression, with the Cl/Fe ratio as the predictor and Cl (specific ion meter) as the outcome variable. This gives a significant result (*F* = 36.42, *P* < 0.01), with *R*^2^ = 0.82 and significant values for the intercept and slope coefficients. The 95% confidence intervals contain the majority of the data points (Fig. 8), but analysis of many more objects from these and other sites is required to validate and extend the model to improve estimation of upper and lower limits for Cl content from Cl/Fe ratios.

The underestimation of the Cl/Fe ratio compared to specific ion meter measurements for BWB83 objects may have a range of causes. Inaccuracies in the specific ion meter normally result from inadequate calibration or the presence of interfering ions (Rundle 2011). Previous measurements on objects from the same site using the same calibration protocols have provided Cl readings in the 30–100 ppm range (Rimmer *et al*. 2012), similar to those predicted by PGAA Cl/Fe ratios, suggesting that the technique is capable of measuring Cl in this range. This would indicate that it is the Cl/Fe ratio that is underestimating the Cl content, rather than the specific ion meter overestimating it for these objects.

Assuming the accuracy of the specific ion meter measurements, underestimation of the Cl content by PGAA may be due either to a general problem with measuring low Cl or related to the specific nature of BWB83 objects. The former is unlikely, as USK_001 did not show a similar discrepancy between PGAA and specific ion meter (Fig. 7) despite having low Cl. The latter is the most likely explanation. BWB83 objects typically have a high proportion of metal core and only thin corrosion layers, reflected in the low oxygen consumption rate (Table 1). This composition is different to that of CPF08 objects, which typically contain a much higher proportion of corrosion products. Given two objects with the same total mass and Cl content, an object containing more iron metal will result in a smaller Cl/Fe ratio than an object containing corrosion products, as the denser metal contains more Fe in the same mass. This is most clearly seen by comparing USK_001, which contained no iron metal, with BWB83_163, which contained a high proportion of metal core. The objects have a similar mass, and BWB83_163 contains 0.9 mg more Cl than USK_001 (Table 3), but the Cl/Fe ratio of BWB83_163 is eight times smaller than that of USK_001. The use of neutron tomography to estimate the proportion of metal and corrosion product may aid in more accurate determination of Cl using PGAA (Koleini *et al*. 2012). For the above reasons, PGAA is not the appropriate method to determine the absolute Cl concentrations of samples with unknown oxidation states, and it must be used with caution in objects with large metal cores and only small amounts of corrosion product.

### Distribution of chloride within objects and its relationship to corrosion rate data

There are too few objects in this pilot study to determine whether there are any general patterns in the distribution of Cl in objects. It is notable, however, that the two objects with the highest chloride peaks (CPF08_064 and CPF08_063) are also the two objects that have areas that are fully mineralized, with negligible Cl. It has been previously established that fully mineralized objects, such as USK_001, tend to have low Cl content due to the diffusion of Cl ions out of the object back into the ground (Watkinson 1983). As corrosion ceases when there is no further iron in the object, there are no anodic sites creating a positive charge, freeing Cl ions from their role in the corrosion process and allowing them to migrate out of the object over time. In partially corroded objects during burial, it is possible that Cl could move within the object from mineralized areas to areas with remaining metal that support anodic sites with high concentrations of positive charge as Fe^2+^, rather than diffusing out of the object. This would have the effect of removing Cl from the mineralized areas, and increasing the concentration of Cl in areas of remaining metal. Alternatively, local diffusion of Cl out of the object may occur in these areas or other explanations may be possible. The samples studied here would appear to support the theory of Cl loss from mineralized areas of partially mineralized objects, but further evidence is needed to determine what happens to the Cl.

CPF08_063 did not show the expected evidence of physical damage despite high Cl and measurable corrosion rates. There are a number of factors that could account for this. With thick corrosion layers and deep-seated Cl, visual evidence for corrosion may not have appeared on the surface yet. The oxygen consumption of CPF08_063 was slower for the first 20 days and then increased, which suggests that there may not have been sufficient rapid corrosion during the test period to cause visual evidence on the surface. The corrosion layers around the area of high Cl are relatively thick (see the X-ray radiograph, Fig. 4), which may result in strong physical integrity within them that requires significant post-excavation corrosion before the growth of corrosion products at the metal surface produces stress cracking and lamination. This would obscure or delay visual evidence of corrosion. In the bulk PGAA measurement, CPF08_063 was one of the objects that had high Cl but a lower corrosion rate than other objects with lower Cl (Table 3), which may be readily explained if there were significant amounts of akaganéite present from post-excavation corrosion, binding some of the Cl within its crystal structure, where it is unable to contribute to active corrosion.

This is the first study that has offered data on the distribution of bulk chloride within objects and linked it to X-radiographic data recording the physical condition of the object and images recording visual damage. This offers a significant insight into corrosion processes and their outcomes. Previously, objects have been cut into sections and digested to determine the Cl content of the sections (Rimmer *et al*. 2012), and cross-sectioned and polished to offer two-dimensional analysis that characterized chloride-bearing corrosion products by micro-analytical synchrotron methods to reveal data on Cl location within the transverse profile (Réguer *et al*. 2007). The employment of such analytical techniques here, following the PGAA and PGAI analysis, would have supplemented and complemented the data produced. This combined approach could be usefully employed for a further range of test samples.

**Figure 9:**
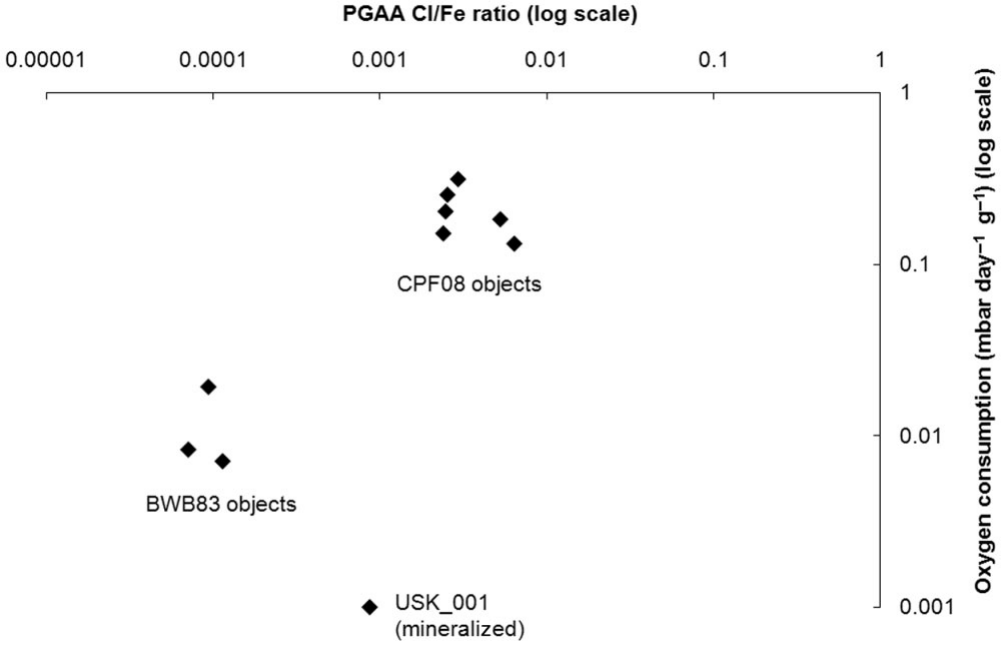
Cl/Fe ratio and oxygen consumption data on log scales. This shows clear discrimination between the groups from different sites on the two measures. The error bars are smaller than the point symbols and are not shown.

Figure 9 shows the relationship between the Cl/Fe ratio and the corrosion rate. The highest Cl contents do not produce the highest corrosion rates, but the three sites are clearly distinguishable into groups based on the two variables (Fig. 9). The variability within each group suggests that a number of other factors are influencing individual corrosion rates, such as the distribution of chloride, the form of the chloride, the degree of mineralization, the thickness of the corrosion layers, the composition of the metal, the object morphology or other unknown variables. The data gathered in this study clearly illustrate that the relationship between the corrosion rate, the chloride level and the visual evidence of corrosion is not straightforward and demands further investigation.

The correlation between the Cl/Fe ratio and the corrosion rate of the objects, as determined by oxygen consumption, is significant on both parametric and non-parametric measures (*r* = −0.58, *P* (one-tailed) < 0.05, *r*_s_ = 0.64, *P* < 0.05, *τ* = 0.42, *P* < 0.05) (Fig. 9). However, a linear regression using the Cl/Fe ratio to predict the corrosion rate does not produce a statistically significant outcome (*P* = 0.08). More data are needed to carry out this type of analysis.

Previous work suggests that the Cl content is a significant predictor of the corrosion rate (Rimmer *et al*. 2013). The use of PGAA data to predict the corrosion rate of objects in a variety of museum environments would be a significant step forward in the use of non-destructive analysis to support conservation planning and preservation strategies. Currently, the Cl contents of museum objects are unknown, and so objects are placed in environments without any prior indication of how rapidly they might corrode. This places them at unknown risk, which can lead to objects beginning to corrode. Even if objects are later stored at low humidity to prevent further corrosion, damage has already occurred and heritage value lost. An accurate method for predicting corrosion behaviour based on chloride levels measured non-destructively by PGAA would improve the assessment of risk for archaeological iron in museum collections and provide an evidence base for storage and management strategies.

Further work to examine whether objects from the same site tend to cluster within particular corrosion ranges could be explored by examining many sites and multiple objects. If a relationship was identified, this could be used to interpret the likely corrosion rate of groups of objects from a particular site by selective PGAA analysis of representative objects from that site, thereby making the data applicable for managing the preservation of hundreds or thousands of objects. This would broaden the application of PGAA, which—due to its cost and rarity—must be limited to material of significant importance or small sample groups. While oxygen consumption can be used to predict the corrosion risk of any iron object, this necessarily requires the object to corrode and thus damage may result. Additionally, objects with reactive species such as iron sulphides that can readily oxidize are difficult to assess using oxygen consumption techniques.

## Conclusions

PGAA and PGAI are excellent techniques for non-destructively analysing the bulk composition and Cl content of archaeological iron objects, because of their high sensitivity for Cl detection. However, to determine the absolute concentration of Cl, it would be necessary to measure directly or calculate the amount of oxygen in the object, which presents some analytical difficulties. Bulk PGAA measurements, expressed as Cl/Fe ratios, easily distinguish between high-Cl and low-Cl objects from different sites and, with incremental analysis using collimators, can reveal the longitudinal distribution of Cl within an individual object. In general, the Cl distribution within objects is highly variable, with areas of high and low Cl in all objects. Areas of high Cl correspond with areas of remaining metal core, visual corrosion phenomena on the surface and attack of the metal core. Detailed imaging of objects, including 3D neutron tomography, is helpful in understanding the chloride distribution, and can be carried out at the same analysis station.

PGAA has significant potential for application in the study of archaeological iron and its corrosion behaviour, in three main areas:

- Compositional data: this paper has focused on Cl, but PGAA produces an elemental analysis across a large range of elements, including trace elements. PGAA is particularly suitable for determining the average composition of heterogeneous materials, which provides additional information to complement surface or spot analyses using other techniques.
- The non-destructive measurement of Cl in whole objects with high sensitivity, which is not currently possible using any other technique. A particular advantage is that PGAA is capable of looking through thick samples, including corrosion layers and metal cores.
- Cl measurements correlate well with the traditional method of measuring Cl. With further work to characterize relationships for different groups of material, PGAA could be used to measure Cl in objects and then predict their corrosion behaviour on the basis of other studies.
- During the analysis, no damage or remaining induced radioactivity is produced in the samples.

Challenges in the use of PGAA for heritage objects include the following:

1. Difficulties in the determination of the absolute concentrations of elements due to the presence of metal core, which makes the calculation of oxygen content based on the full-oxidization assumption inappropriate. Longer data acquisition to detect the otherwise weak oxygen signal may help to solve this issue, but would be significantly more time-consuming.
2. Size limitation: the maximum dimensions of an object that can be measured in the regular sample position of the PGAA and NIPS–NORMA stations at Budapest is 20 × 20 × 40 mm, without dismantling the sample holders. This limits the analysis to relatively small archaeological artefacts. Larger neutron beamlines such as ISIS, designed for engineering structures, may prove suitable for future studies of larger artefacts provided PGAI is available.

## Appendix: Supplier List

### Chemicals

AnaLar nitric acid, HNO_3_ and AnaLar sodium hydroxide, NaOH, Fisher Scientific UK Ltd, Bishop Meadow, Loughborough, Leicestershire, LE11 5RG, UK. http://www.fisher.co.uk

### Equipment

Binder KBF240 constant climate chamber with humidity, supplied by Lab Mode Ltd, 409 Centennial Avenue, Elstree, Borehamwood, WD6 3TN, UK. http://www.labmode.co.uk

Radiometer Analytical PHM250 specific ion meter, ISE25CL chloride-specific electrode, REF621 chloride-free Hg/HgSO_4_ reference electrode, Radiometer Analytical, 72 rue d′Alsace, 69627 Villeurbanne CEDEX, Lyon, France. http://www.radiometer-analytical.com

OxyMini fibre-optic oxygen meter with sensor spots, World Precision Instruments Ltd, 1 Hunting Gate, Hitchin, Hertfordshire SG4 0TJ, UK. http://www.wpi-europe.com

Self-indicating (orange–green) silica gel beads, supplied by GeeJay Chemicals Ltd, 1 Beamish Close, Sandy, Bedfordshire, SG19 1SD, UK. http://www.geejaychemicals.co.uk

Mason-Ball 250 ml preserving jars and lids, supplied by Golda′s Kitchen Inc., 2885 Argentia Rd Unit 6, Mississauga, Ontario, L5N 8G6, Canada. http://www.goldaskitchen.com

Madgetech RHTemp101A humidity/temperature dataloggers, supplied by LoggerShop Technology, 189 Ashley Road, Poole, Dorset, BH14 9DL, UK. http://www.loggershop.co.uk
